# Intra‐annual growing season climate variability drives the community intra‐annual stability of a temperate grassland by altering intra‐annual species asynchrony and richness in Inner Mongolia, China

**DOI:** 10.1002/ece3.9385

**Published:** 2022-10-04

**Authors:** Ze Zhang, Tiejun Bao, Yann Hautier, Jie Yang, Zhongling Liu, Hua Qing

**Affiliations:** ^1^ Ministry of Education Key Laboratory of Ecology and Resource Use of the Mongolian Plateau Inner Mongolia University Hohhot China; ^2^ Inner Mongolia Key Laboratory of Grassland Ecology School of Ecology and Environment, Inner Mongolia University Hohhot China; ^3^ Ecology and Biodiversity Group, Department of Biology Utrecht University Utrecht Netherlands

**Keywords:** climate variability, dominant species intra‐annual stability, functional group intra‐annual stability, intra‐annual species asynchrony, intra‐annual species richness, long‐term observation

## Abstract

Understanding the factors that regulate the functioning of our ecosystems in response to environmental changes can help to maintain the stable provisioning of ecosystem services to mankind. This is especially relevant given the increased variability of environmental conditions due to human activities. In particular, maintaining a stable production and plant biomass during the growing season (intra‐annual stability) despite pervasive and directional changes in temperature and precipitation through time can help to secure food supply to wild animals, livestock, and humans. Here, we conducted a 29‐year field observational study in a temperate grassland to explore how the intra‐annual stability of primary productivity is influenced by biotic and abiotic variables through time. We found that intra‐annual precipitation variability in the growing season indirectly influenced the community intra‐annual biomass stability by its negative effect on intra‐annual species asynchrony. While the intra‐annual temperature variability in the growing season indirectly altered community intra‐annual biomass stability through affecting the intra‐annual species richness. At the same time, although the intra‐annual biomass stability of the dominant species and the dominant functional group were insensitive to climate variability, they also promoted the stable community biomass to a certain extent. Our results indicate that ongoing intra‐annual climate variability affects community intra‐annual biomass stability in the temperate grassland, which has important theoretical significance for us to take active measures to deal with climate change.

## Graphical abstract

Intra‐annual growing season precipitation variability and temperature variability indirectly influenced the community intra‐annual biomass stability by negative effect on intra‐annual species asynchrony and intra‐annual species richness, respectively. ; Intra‐annual biomass stability of dominant species and dominant functional group were insensitive to climate variability, they also promoted the stable community biomass to a certain extent.

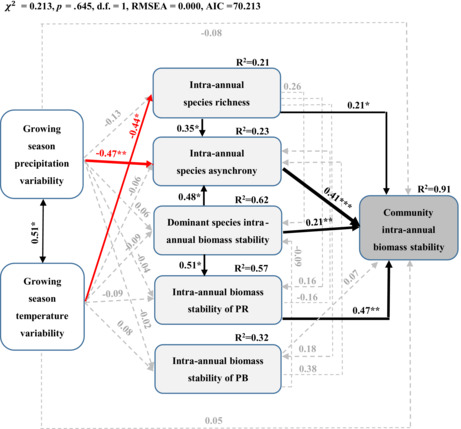

## 1 INTRODUCTION

Stability is one of the most fundamental and studied properties of an ecosystem (Hautier et al., 2014; Ma et al., 2017; Xu et al., 2015). In particular, the stability of ecosystem primary productivity through time gives us information about the ability of an ecosystem to provide reliable biomass despite environmental fluctuations (Craven et al., 2018; Jiang et al., 2009; Pimm, 1984). Grasslands are one of the most widely distributed ecosystems worldwide (Häyhä & Franzese, 2014), providing not only key habitat for biodiversity but also other important ecosystem functions and services to humanity (Isbell et al., 2009). Understanding the processes that influence the temporal stability of grasslands' productivity is a pressing issue in ecology, especially given its vulnerability to anthropogenic and climatic changes (Ives & Carpenter, 2007).

Profound climate variability such as global warming and changes in precipitation patterns (IPCC, 2013; Min et al., 2011; Orlowsky & Seneviratne, 2012; Putnam & Broecker, 2017) are affecting the biodiversity and functioning of grassland ecosystems (Kardol et al., 2010). Previous studies in grasslands have shown that increased precipitation variability in the growing season resulted in a decline in aboveground net primary productivity (ANPP) by delaying plant phenology and limiting leaf expansion as well as reducing tillering, root range and microbial biomass carbon (Chen et al., 2020; Craine et al., 2012; De Micco & Aronne, 2012; Robinson et al., 2013; Yang et al., 2016). Species richness and dominant species abundance of the community decreased with the increased temperature variability in the growing season (Ma et al., 2017; Yang et al., 2017; Zhang et al., 2018). Meanwhile, studies also found that non‐growing season (winter) climate resources could stimulate plant production by increasing soil nutrients and water supply at the beginning of the growing season (Li et al., 2020; Schimel et al., 2004; Semenchuk et al., 2015). Additionally, different plant functional groups might respond differently to intra‐annual climate variability based on differences in their physiology and life history (Huenneke et al., 2002; Mulhouse et al., 2017; Munson et al., 2014). For example, algorithmic analysis based on seasonal water availability showed that the relative biomass of C_3_/C_4_ grasses was determined by the allocation of effective water and temperature between C_3_ grasses and C_4_ grasses during the growing season (Winslow et al., 2003). Decreased precipitation in the early growing season mainly resulted in decreased ANPP of perennial grass, whereas decreased precipitation in the late growing season primarily resulted in decreased ANPP of perennial forbs (Zhang et al., 2020). These changes in productivity through time may translate into lower stability of productivity in response to climate change.

However, changes in temperature and precipitation might be notably stronger at the seasonal rather than annual scale (Donat et al., 2016; Zhang et al., 2018), suggesting that the intra‐annual variability of temperature and precipitation may be the main driver of grassland stability. Previous studies have primarily focused on the temporal stability of the community biomass measured at one time during the growing season (usually at peak biomass production) each year over multiple years (inter‐annual stability) (Chi et al., 2019; Ma et al., 2017; Xu et al., 2015). But whether the intra‐annual variability of temperature and precipitation affect the intra‐annual biomass stability of community remains unknown. This is important given that community intra‐annual biomass stability governs secure food supply to wild animals, livestock, and humans.

Theoretical and empirical evidence suggested that the temporal stability of ecosystems was influenced by multiple underlying mechanisms (Huang et al., 2020; Ma et al., 2017). First, a higher number of plant species usually resulted in a higher stability of biomass production (Hautier et al., 2014). Thus, a reduction in plant diversity in response to climate change may result in a reduction in stability (Campbell et al., 2011; Hautier et al., 2014; Zhang et al., 2018). Second, community stability may be driven primarily by the stability of dominant species and/or functional groups, especially when dominant species and/or functional groups account for a considerable proportion of community biomass (Hillebrand et al., 2008; Huang et al., 2020; Ma et al., 2022). Third, asynchronous dynamics among species may contribute largely to stabilizing community properties against environmental changes (Loreau & de Mazancourt, 2013; Valencia et al., 2020). Species asynchrony usually increased with increasing species richness (Hector et al., 2010). As a result, changes in temperature and precipitation may affect community stability by changing asynchronous dynamics among species which directly or indirectly are induced via changes in species richness (Hallett et al., 2014; Hautier et al., 2020; Sasaki et al., 2019). To summarize, intra‐annual variability of temperature and precipitation may affect community stability by changing species richness (Arnone III et al., 2011; Klein et al., 2004), dominant species stability (Xu et al., 2015), functional group stability (Huang et al., 2020) and species asynchrony (Zhang et al., 2018; Zhou et al., 2019). However, these characteristics are not isolated, and how they affect each other and contribute to temporal stability needs to be further studied.

Long‐term monitoring can reveal the long‐term dynamic of plant communities in response to climate variability, and the relationship between community stability with long‐term climate variability (Bai et al., 2004; Li et al., 2015; Zhou et al., 2019). Here, we collected long‐term monthly data on community above‐ground biomass, species composition, species richness, and climate data of a temperate grassland from 1981 to 2011 in northern China, and analyzed the effect of intra‐annual temperature and precipitation variability during growing and non‐growing seasons on community intra‐annual biomass stability. To our knowledge, this is the first study to investigate the relationship of intra‐annual climate variability with intra‐annual biomass stability of the grassland ecosystem. We hypothesized that plant community would be more unstable when the intra‐annual climate is more unstable because (1) higher intra‐annual climate variability reduces the intra‐annual species richness and asynchrony, (2) higher intra‐annual climate variability reduces the intra‐annual biomass stability of dominant species/functional group.

## 2 MATERIALS AND METHODS

### 2.1 Study site

The investigation was conducted at Inner Mongolia Grassland Ecosystem Research Station, which is located in a temperate grassland in the Inner Mongolia, China (116.8°E, 43.5°N, 1179 m a.s.l.) (Appendix S1). The study site has a temperate continental climate. The long‐term (1981–2011) mean annual temperature (MAT) was 0.78°C, with the minimum mean monthly temperature being −21.3°C in January and the maximum mean monthly temperature being 19.3°C in July (Appendix S1). The long‐term annual precipitation (AP) was 330.1 mm, of which 85% falling in the growing season (from May to September). Over the period 1981–2011, MAT showed an increasing trend with a rate of 0.06°C/year, while AP fluctuated between 166.1 and 507 mm (Appendix S1). The study site was dominated by two perennial rhizome grasses species *Leymus chinensis* and *Agropyron cristatum* with the relative abundance being 25.5 ± 4.2% and 7.1 ± 1.6% respectively, and two perennial bunchgrass species *Stipa grandis* and *Achnatherum sibiricum* with the relative abundance separately being 19.1 ± 3.6% and 11.2 ± 2.7%. These species were the most widely distributed species at the study site and accounted for 62.9 ± 10.1% of the above‐ground biomass total. According to Chinese classification, the soil type of study site was chestnut soil, with an average bulk density of 0–20 cm soil layer being 1.29 g/cm^3^ and a pH of 7.68 (Yuan et al., 2005).

### 2.2 Sample site design

The study site consisted of a relatively flat and even area of 600 m by 300 m, fenced since 1979 to prevent grazing by large animals (Li et al., 2015). In 1981, the area was equally separated into 10 replicate blocks (60 × 300 m each). Community aboveground biomass was surveyed in the middle of every month throughout the growing season (from May to September) of each year by clipping green parts of all vascular plants above the soil surface within a 1 × 1 m quadrat over 1981–2011. The quadrat was randomly located within each block, and for each survey the location of the quadrat was marked to avoid setting up the quadrat at the same site. Other areas in each block that were not harvested remained undisturbed. After harvesting, all living vascular plants were sorted into the species, and oven‐dried at 65°C to a constant weight. Intra‐annual species richness within each block was calculated as the total number of species recorded from May to September of each year. All species were classified into five plant functional groups primarily on the basis of life forms (Bai et al., 2004): perennial rhizome grass (PR), perennial bunchgrasses (PB), perennial forbs (PF), shrubs and semi‐shrubs (SS), and annuals and biennials (AB). Functional group biomass was determined as the biomass sum of all the species in each functional group. Hence, community aboveground biomass was estimated for 1450 quadrats (i.e., 1 quadrat × 10 blocks per month × 5 months per year × 29 years = 1450 quadrats) excluding missing data from the years 1995 and 1996 (Ma et al., 2010).

### 2.3 Climate data

The monthly mean temperature and monthly cumulative precipitation data were collected from the weather station situated about 9 km from the study site. It has been found that plant phenology and community aboveground biomass in the temperate grassland were affected by temperature and precipitation fluctuations during the growing season and non‐growing season (Bai et al., 2004; Li et al., 2015; Li et al., 2019; Zhang et al., 2020). So, in this case, we calculated the intra‐annual temperature and precipitation variability in the growing season (May to September) and non‐growing season (October of the previous year to April of the current year) with the calculation formula of *σ*/*μ* × 100, where σ and μ were separately the standard deviation and mean of temperature or precipitation in the growing season or non‐growing season in each year from 1981 to 2011.

### 2.4 Statistical analysis

Similar to climate variability, the intra‐annual stability of community biomass was calculated annually as *μ*/*σ* (Ma et al., 2017), where *μ* was the intra‐annual mean community biomass (from May to September), and *σ* was its standard deviation. The intra‐annual stability of dominant species (*L. chinensis*, *A. cristatum*, *A. sibiricum* and *S. grandis*) and functional group biomass were also calculated annually using the same method. A higher value of community intra‐annual biomass stability means a lower intra‐annual variability of community biomass (Lehman & Tilman, 2000).

Intra‐annual species asynchrony, which refers to the asynchronous response of species to environmental fluctuations from May to September in each year (Loreau & De Mazancourt, 2008), was calculated as:

$$1-\varphi_{x}=1-\frac{\sigma^{2}}{{\left(\sum_{l=1}^{T}\sigma_{l}\right)}^{2}}$$

where $\varphi_{x}$ was intra‐annual species synchrony, $\sigma^{2}$ and $\sigma_{l}$ were the variance of intra‐annual community mean biomass (from May to September), and the standard deviation of biomass of species *l* in a plot with *T* species. Intra‐annual species asynchrony ranges between 0 and 1, and higher values correspond to higher asynchronous dynamics between species within the community, and vice versa.

The annual change rates of intra‐annual precipitation and temperature variability in growing/non‐growing seasons were calculated by using the slope of a simple linear regression equation of intra‐annual precipitation or temperature variability with years. Independent samples *t* tests were used to compare significant differences in intra‐annual precipitation/temperature variability between the growing and non‐growing seasons. In order to investigate inter‐annual variations in intra‐annual community biomass, intra‐annual species richness, intra‐annual species asynchrony, and intra‐annual biomass stability of dominant species/functional group/community across 1981–2011, we used the slope of a simple linear regression between these variables and years as an indicator of their trends over time. Simple linear regressions were also used to assess how intra‐annual precipitation/temperature variability in growing/non‐growing season, intra‐annual community biomass, intra‐annual species richness, intra‐annual species asynchrony, and intra‐annual biomass stability of dominant species/functional group related to community intra‐annual biomass stability. SPSS 19.0 software package was used for all the analysis.

To address mechanisms determining community intra‐annual biomass stability in response to climate variability, structural equation modeling (SEM) was used to assess the effects of intra‐annual precipitation and temperature variability in growing and non‐growing seasons on community intra‐annual biomass stability through intra‐annual species richness, intra‐annual species asynchrony, and intra‐annual biomass stability of functional group and dominant species. We constructed an a priori model (Appendix S1) based on the known effects and potential relationships among the drivers of community intra‐annual stability. In the model, we assumed that climate variability had the potential to alter community intra‐annual biomass stability directly, as well as indirectly through changing intra‐annual species richness, intra‐annual species asynchrony, and dominant species or functional group intra‐annual biomass stability. Based on regression weight estimation, the initial model was simplified and non‐significant path and state variables were eliminated, and the final model contained only statistically significant standardized paths that could not be rejected (Appendix S1). The accuracy of the model was confirmed using a Chi‐squared test, the Akaike Information Criterion (AIC), and the root‐mean‐square errors of approximation (RMSEA). The model has a good fit when Chi‐squared test $\chi^{2}\geq 0,$ *p* > .05, and 0 ≤ RMSEA ≤ 0.05. Structural equation model analysis was performed by AMOS 22.0.

## 3 RESULTS

### 3.1 Inter‐annual change in climate intra‐annual variability

**FIGURE 1:**
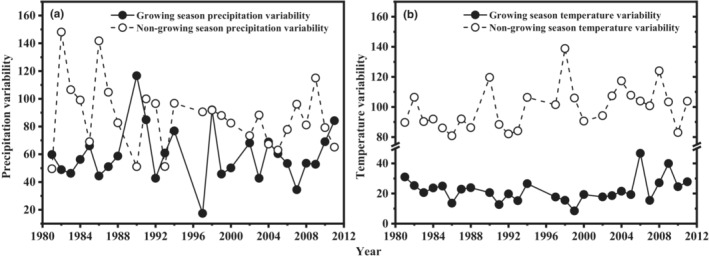
Intra‐annual precipitation variability (a) and temperature variability (b) in growing and non‐growing seasons from 1981 to 2011.

Intra‐annual variability of precipitation and temperature during the growing and non‐growing seasons did not show any temporal trends from 1981 to 2011 (Figure 1). Compared with those of growing season, intra‐annual variability of precipitation (Figure 1a; *F*_1,29_ = 38.2, *p* ˂ .001) and temperature (Figure 1b; *F*_1,29_ = 65.2, *p* ˂ .001) in the non‐growing season were stronger.

### 3.2 Inter‐annual variation of intra‐annual community biomass, species richness, and asynchrony

**FIGURE 2:**
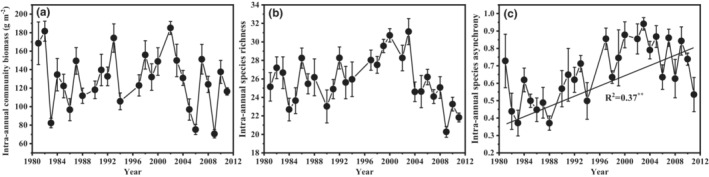
Changes in the intra‐annual (a) community biomass (the mean from May to September), (b) species richness and (c) species asynchrony from 1981 to 2011 (*n* = 10, with standard deviation).

From 1981 to 2011, both intra‐annual community biomass (Figure 2a) and intra‐annual species richness (Figure 2b) did not show obvious change trend, while the intra‐annual species asynchrony increased significantly with time (Figure 2c; *F*_1,29_ = 9.7, *p* = .001, *R* ^2^ = .37). Across these 29 years, the mean intra‐annual community biomass during the growing season was 130.19 g/m^2^ with a range between 70.60 and 185.03 g/m^2^ (Figure 2a), and the mean intra‐annual species richness was 25.9 species, with a range between 20.3 and 31.1 species (Figure 2b).

### 3.3 Variation of intra‐annual biomass stability of community, functional group and dominant species

**FIGURE 3:**
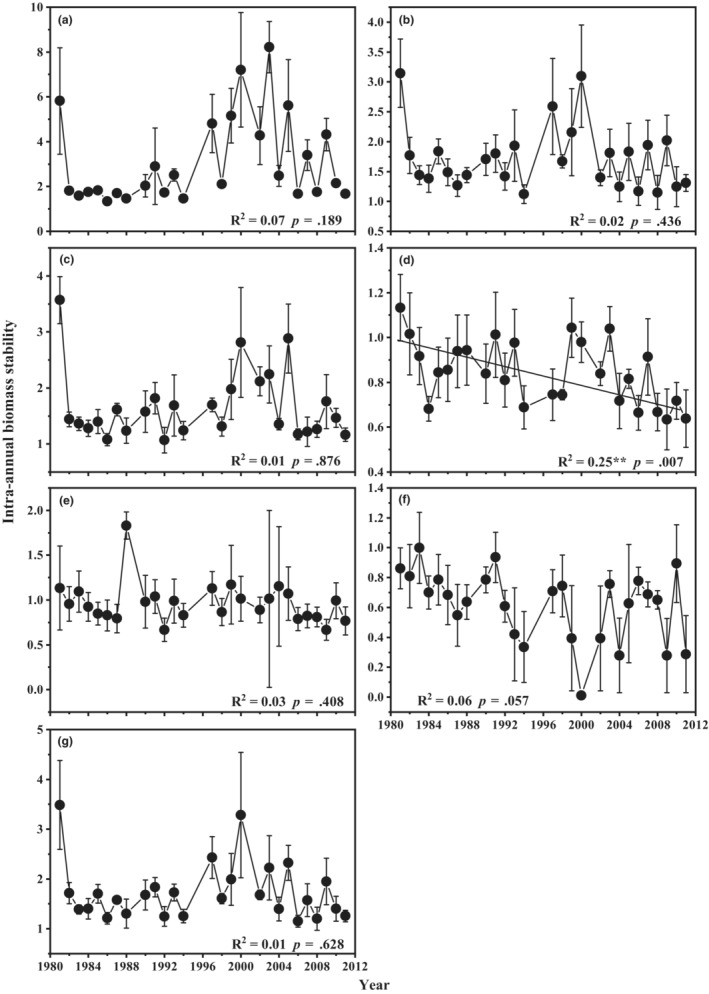
Variation of intra‐annual biomass stability of (a) community, (b) functional group PR, (c) functional group PB, (d) functional group PF, (e) functional group SS, (f) functional group AB, and (g) dominant species across 1981 to 2011 analyzed by simple linear regression. (*n* = 10, with standard deviation). The black solid line was a significant regression line. Asterisks indicate a significant changes (*p < .05, **p < .01). PR, perennial rhizome grass; PB, perennial bunchgrasses; PF, perennial forbs; SS, shrubs and semi‐shrubs; AB, annuals and biennials.

From 1981 to 2011, there was no significant change trend in intra‐annual biomass stability of community (Figure 3a), functional group PR (Figure 3b), PB (Figure 3c), SS (Figure 3e) and AB (Figure 3f), and dominant species (Figure 3g). However, the intra‐annual biomass stability of functional group PF showed a significant decreasing during these 29 years (Figure 3d; *F*_1,29_ = 14.6, *p* = .007, *R* ^2^ = .25).

### 3.4 The relationship of community intra‐annual biomass stability with climatic and biotic factors

**FIGURE 4:**
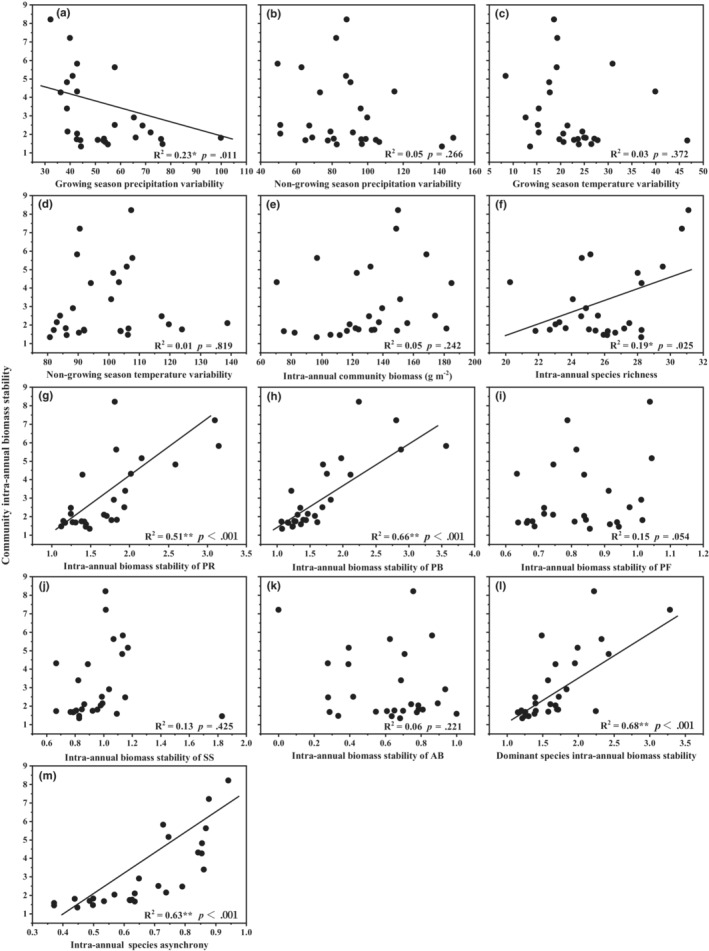
Community intra‐annual biomass stability in relation to (a) growing season precipitation variability, (b) non‐growing season precipitation variability, (c) growing season temperature variability, (d) non‐growing season temperature variability, (e) intra‐annual community biomass, (f) intra‐annual species richness, (g) intra‐annual biomass stability of functional group PR, (h) intra‐annual biomass stability of functional group PB, (i) intra‐annual biomass stability of functional group PF, (j) intra‐annual biomass stability of functional group SS, (k) intra‐annual biomass stability of functional group AB, (l) intra‐annual biomass stability of dominant species, and (m) intra‐annual species asynchrony, analyzed by simple linear regression. The black solid lines were significant regression lines. Asterisks indicate significant correlation (*p < .05, **p < .01). PR, perennial rhizome grass; PB, perennial bunchgrasses; PF, perennial forbs; SS, shrubs and semi‐shrubs; AB, annuals and biennials.

Intra‐annual biomass stability of the community was significantly negatively correlated with the intra‐annual variability of precipitation in the growing season (Figure 4a; *p* = .011, *R* ^2^ = .23), but had significantly positive relationships with intra‐annual species richness (Figure 4f; *p* = .025, *R* ^2^ = .18), species asynchrony (Figure 4m; *p* ˂ .001, *R* ^2^ = .63), and biomass stability of functional group PR (Figure 4g; *p* ˂ .001, *R* ^2^ = .51), PB (Figure 4h; *p* ˂ .001, *R* ^2^ = .66) and dominant species (Figure 4l; *p* ˂ .001, *R* ^2^ = .53). No significant relationship was found between community intra‐annual biomass stability and precipitation variability in the non‐growing season (Figure 4b), temperature variability in the growing and non‐growing season (Figure 4c,d), intra‐annual community biomass (Figure 4e), and intra‐annual biomass stability of functional group PF (Figure 4i), SS (Figure 4j) and AB (Figure 4k).

### 3.5 Direct and indirect effects of climatic and biotic factors on the intra‐annual stability of community

**FIGURE 5:**
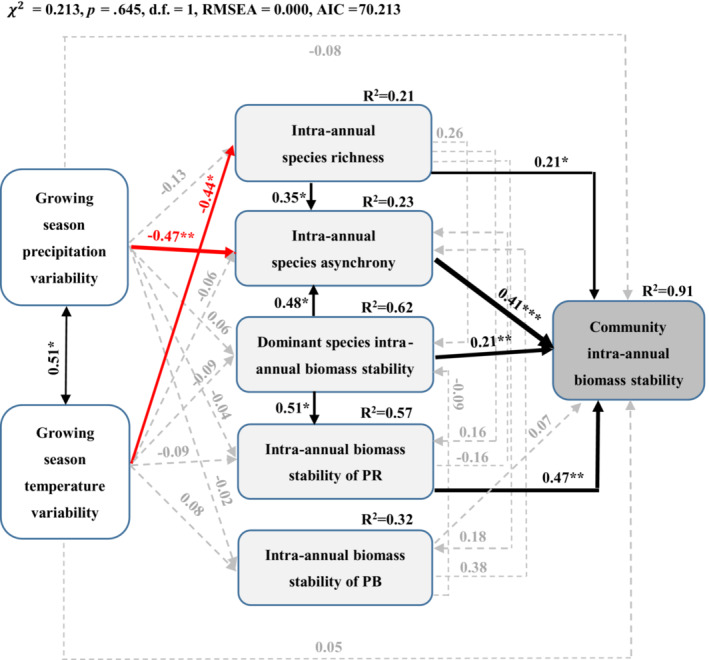
Structural equation model of growing season precipitation (temperature) variability, intra‐annual species richness and asynchrony, dominant species intra‐annual biomass stability and intra‐annual biomass stability of PR (perennial rhizome grass) and PB (perennial bunchgrasses) on community intra‐annual biomass stability. Black and red arrows represented significant positive and negative pathways respectively, and gray dashed arrows indicated nonsignificant pathways. Arrow width was proportional to the strength of the relationship. Numbers adjacent to arrows were standardized path coefficients and indicated the effect size of the relationship. The proportion of variance explained (*R* ^2^) appeared alongside response variables in the model, and asterisks indicated statistical significance (*p < .05, **p < .01, ***p < .001).

SEM analysis showed that intra‐annual species richness and asynchrony, dominant species intra‐annual biomass stability and intra‐annual biomass stability of PR had direct positive effects on community intra‐annual biomass stability (Figure 5). Increased intra‐annual species richness and intra‐annual biomass stability of dominant species also indirectly improved the community intra‐annual biomass stability by promoting intra‐annual species asynchrony. Dominant species intra‐annual biomass stability was also found to affect community intra‐annual biomass stability indirectly by changing the intra‐annual biomass stability of PR. In addition, the increased intra‐annual precipitation and temperature variability in the growing season indirectly reduced community intra‐annual biomass stability by weakening intra‐annual species asynchrony and species richness respectively (Figure 5).

## 4 DISCUSSION

Based on 29 years of field observation data, we found that growing season precipitation variability indirectly influenced the community intra‐annual biomass stability by a negative effect on intra‐annual species asynchrony. Meanwhile, growing season temperature variability altered intra‐annual community biomass stability by negatively affecting the intra‐annual species richness, supporting our hypothesis (1). However, contrary to our hypothesis (2), we did not find that the intra‐annual biomass stability of the dominant species or functional groups changed with the change of intra‐annual climate variability, but they promoted the stable community biomass to a certain extent.

Climate change, including increased climate variability as well as changed distribution patterns of temperature or precipitation, has had an important impact on community composition and species dynamics of the Inner Mongolia temperate grassland over the past several decades (Li et al., 2015; Ma et al., 2010; Zhang et al., 2020), which may in turn affect the community biomass stability. On the inter‐annual scale, studies have found that the community biomass stability was affected by inter‐annual climate variability (Gilbert et al., 2020; Zhang et al., 2018). However, on a seasonal scale, this study found that the intra‐annual variability of precipitation in the growing season did not directly affect the community intra‐annual biomass stability, which may be due to the lower variability of precipitation among the growing seasons relative to the years. During the 29 years of observation period, the mean growing season precipitation variability was about 51.45, which was less than the inter‐annual variation of precipitation variability in the study area (approximately 67; Zhang et al., 2018), indicating the lower precipitation variability, the weaker its direct effect on the community intra‐annual biomass stability.

However, although it had no direct effect, the intra‐annual variability of precipitation in the growing season indirectly affected the community intra‐annual biomass stability by reducing intra‐annual species asynchrony (Figure 5). Species asynchrony is a common feature of ecological communities (Blüthgen et al., 2016; Gonzalez & Loreau, 2009), which is a general mechanism to maintain the community stability (Ma et al., 2017; Xu et al., 2015), and can be dependent on asynchronous species responses to environmental fluctuations (Douda et al., 2018; Ives & Carpenter, 2007; Loreau & De Mazancourt, 2008). In our study, the intra‐annual species asynchrony determined the community intra‐annual biomass stability to a great extent (Figure 5). The prediction of species asynchrony response to precipitation variability is still controversial (Gilbert et al., 2020; Ma et al., 2017; Xu et al., 2015; Zhang et al., 2018). Our long‐term observation study was consistent with the findings of Gilbert et al. (2020) that precipitation variability indirectly influenced stability through asynchronous responses, supporting the theory that low or extreme climate variation will limit asynchronous dynamics by reducing the potential for temporal niche partitioning (Adler & Drake, 2008). Extreme events caused by climate fluctuations (such as drought, summer frost or heat wave) may lead to temporal dynamic convergence (i.e., synchronize) of species, which probably results in correlated mortality among species (Hoover et al., 2014). Therefore, a negative correlation between precipitation variability and species asynchrony could be found, just as our results showed (Figure 5). Water is the main limiting factor of productivity in arid and semi‐arid grassland (Sala et al., 2012), which underpins photosynthesis, cell structure, the transport of nutrients and, ultimately, carbon balance (Fang et al., 2001; Yang et al., 2011). In the study area observed here, the perennial grasses *L. chinensis* and *A. cristatum* and the perennial forbs *Potentilla bifurca* and *P. tanacetifolia* contributed to community biomass in the early growing season, while the perennial forbs *Axyria amaranthoides*, *Iris tenuifolia* and *Allium tenuissimum* as well as rare annuals and biennials *Orostachys fimbriatus* and *Dysphania aristata* with high growth rate mainly took advantage of precipitation in the late growing season, and were very sensitive to climate variability (Appendix S1; Bai et al., 2004; Li et al., 2015; Zhang et al., 2020). These species showed obvious intra‐annual asynchronous response to precipitation in the growing season, which stabilized the intra‐annual community biomass to a great extent. Therefore, in our study area, growing season precipitation variability affected the community structure and stability of the grassland ecosystem, which is consistent with previous studies (Bai et al., 2004; Chen et al., 2020; Robinson et al., 2013; Zhang et al., 2020).

Similarly, we also found no direct relationships between temperature variability in the growing season or non‐growing season and community intra‐annual biomass stability, which might be due to the asymmetry of the effects of daytime and nighttime temperature variability on community intra‐annual stability, consistent with results of climate control experiments on temperate grassland and alpine meadow (Ma et al., 2017; Yang et al., 2017; Zhou et al., 2019). However, we found that growing season temperature variability indirectly affected community intra‐annual biomass stability by negatively influencing species richness. Significant temperature variability could cause serious environmental restrictions, which might reduce the reproductive capacity of plants and cause physiological failure (such as seedling establishment failure), and thus lead to high mortality of plants and reducing species richness (Andrus et al., 2018; Reyer et al., 2013).

Several field observations and theoretical models have suggested that community stability may increase with increasing species richness (Gross et al., 2014; Jiang et al., 2009; Mougi & Kondoh, 2012). Consistent with the positive diversity‐stability relationship often reported in experimental studies (Loreau & de Mazancourt, 2013; Tredennick et al., 2017), in the present study, intra‐annual species richness directly contributed to community intra‐annual biomass stability (Figure 5), indicating that restoring and protecting biodiversity can provide sustainable ecosystem functioning (Adler, 2011; Grace, 2007). Diversity‐dependent stability mechanisms have been found to mainly include overyielding (i.e., positive diversity‐productivity relationships; Cardinale et al., 2006; Gross et al., 2014; Hautier et al., 2020), complementarity effect (species asynchrony; Hautier et al., 2014; Loreau & De Mazancourt, 2013; Tredennick et al., 2017) and the portfolio effect (Doak et al., 1998; Thibaut & Connolly, 2013). In this study, we found that the positive relationship between intra‐annual species richness and asynchrony, rather than overyielding (Appendix S1), maintained community intra‐annual biomass stability (Figure 5), indicating that compensation effect among species can promote community intra‐annual biomass stability. In particular, there was a significant increasing trend in both community intra‐annual species asynchrony (Figure 2c; *F*_1,19_ = 13.4, *R* ^2^ = .44, *p* = .002) and intra‐annual species richness (Figure 2b; *F*_1,19_ = 21.4, *R* ^2^ = .56, *p* ˂.001; average increase rate of 0.27 species per year) over the period 1981–2003. In the study site, perennial forbs were the main contributors to the total species richness of the community (58.12 ± 7.96%; Appendix S1). With the appearance of more perennial forbs, such as *Silene aprica*, *Linum perenne*, *Astragalus galactites* and *Medicago ruthenica*, the increased species richness enabled species to develop a wider range of niches, which further significantly contributed to intra‐annual species asynchrony (*F*_1,19_ = 5.8, *R* ^2^ = .25, *p* = .028). The insurance hypothesis states that species richness can increase the possibility of species with different responses to environmental conditions and disturbances in the community, and results in compensation (asynchrony) among species, which increases the stability of the community (Craven et al., 2018; Hautier et al., 2014; Hector et al., 2010). Meanwhile, temperature variability in the growing season may weaken the diversity‐dependent species asynchrony by changing ecological processes such as plant functional traits, phenology and litter decomposition, and then affects the stability of the community (Cheng et al., 2021; Shaw et al., 2022; Shen et al., 2011).

Ecosystems are largely controlled by the characteristics of the dominant species, i.e., the mass ratio hypothesis (Grime, 1998), which may even constrain the effect of species diversity on biomass stability (Wang et al., 2020; Wayne et al., 2007). In our study, we found that the intra‐annual biomass stability of dominant species contributed to community intra‐annual biomass stability, reinforcing these ideas (Sasaki & Lauenroth, 2011; Wilsey et al., 2014; Xu et al., 2015). In addition, an important finding of our study was that intra‐annual biomass stability of dominant functional group perennial rhizome and perennial bunchgrass were also the important contributors to community intra‐annual biomass stability. In our study area, the biomass of functional group perennial rhizome and perennial bunchgrass accounted for 35.5% and 32.3% of the community total biomass respectively, so they were the two dominant functional group in the study area and stabilized community productivity to a large extent (Appendix S1). However, it is noteworthy that we did not find impacts of intra‐annual climate variability on the intra‐annual biomass stability of dominant species and dominant functional group (Figure 5), which may be caused by the relative insensitivity of the dominant species or dominant functional group to environmental changes. Dominant species (perennial rhizome grasses *L. chinensis* and *A. cristatum*, and perennial bunchgrass *S. grandis* and *A. sibiricum*) in the study site contributed 92.8% of the biomass of the dominant functional group, so their response to climate change greatly affects the response of the dominant functional group to climate change. For a long time, the dominant species in this study area have formed a series of traits to adapt to climate change, so they showed stable biomass under changing climate. Such as, *L. chinensis* can obtain nutrition through developed roots, and have higher plant height and larger specific leaf area, which enables them to obtain more sunlight to cope with the changing climate environment (Yang et al., 2011; Zhang et al., 2020). *S. grandis* has a lower level of lipid peroxidation in leaves, which can protect it from oxidative damage and maintain stable productivity under heat stress, drought stress and their interactive conditions (Song et al., 2016). In addition, compensatory dynamics among different species or functional groups have been proposed as an important mechanism for community stability (Bai et al., 2004; Liu et al., 2018), which may be due to the different use of seasonal precipitation by species or functional group (Hovenden et al., 2014; Li et al., 2015; Zhang et al., 2020). In our study area, there were significant biomass complementary effects between perennial rhizome grasses and perennial bunchgrass, perennial rhizome grasses and perennial forbs, perennial forbs and annuals‐biennials, as well as dominant species *L. chinensis* and *S. grandis* (Bai et al., 2004; Li et al., 2015), which maintained intra‐annual community biomass stability.

In addition, we found no direct or indirect effect of non‐growing season climate variability on community intra‐annual biomass stability (Figure 4). Although studies have shown that the non‐growing season climate resources could stimulate plant production by increasing soil nutrients and water supply at the beginning of the growing season (Schimel et al., 2004; Semenchuk et al., 2015), However, for our study area, plant growth mainly depends on precipitation and temperature in the growing season, and no evidence has been found that the community productivity had a strong response to the legacy effect of soil moisture or nutrients from the non‐growing season (Leizeaga et al., 2021; Peng et al., 2021).

Based on a long‐term study spanning 29 years of observation, our study provides a new practical basis for ongoing intra‐growing season climate variability significantly affecting community intra‐annual biomass stability by influencing intra‐annual species richness and asynchrony, suggesting that intra‐annual climate variability has a negative impact on ecosystem functioning. Our findings elucidated the potential influencing mechanism of intra‐annual climate variability on community intra‐annual temporal stability of temperate grassland in Inner Mongolia, China. However, in future coordinated multi‐sites, larger‐scale studies in a variety of ecosystems are needed to test whether our findings are universal.

## AUTHOR CONTRIBUTIONS

**Ze Zhang:** Conceptualization (lead); formal analysis (lead); methodology (equal); writing – original draft (lead); writing – review and editing (equal). **Tiejun Bao:** Conceptualization (equal); investigation (equal); resources (lead). **Yann Hautier:** Conceptualization (equal); methodology (lead); writing – review and editing (equal). **Jie Yang:** Funding acquisition (lead); resources (equal); supervision (lead). **Zhongling Liu:** Investigation (lead); resources (equal); supervision (equal). **Hua Qing:** Conceptualization (equal); data curation (lead); formal analysis (equal); methodology (equal); writing – review and editing (lead).

## CONFLICT OF INTEREST

The authors declare no competing interests.

## DATA AVAILAVILITY STATEMENT

Data available from the Dryad Digital Repository: Dryad, Dataset, https://doi.org/10.5061/dryad.hmgqnk9k9.

## Supporting information


Appendix S1
