# Supplementary material for: Intra‐annual growing season climate variability drives the community intra‐annual stability of a temperate grassland by altering intra‐annual species asynchrony and richness in Inner Mongolia, China
Source: Ecol Evol. 2022 Oct 4;12(10):e9385. doi: 10.1002/ece3.9385 (PMC9532246; doi:10.1002/ece3.9385)
Supplement: Supplementary file 1 — Appendix S1 [file ECE3-12-e9385-s001.docx]

**Appendix**

**Figure A1.** Location of sampling site (Inner Mongolia Grassland Ecosystem Research Station) of study region in Inner Mongolia, China.


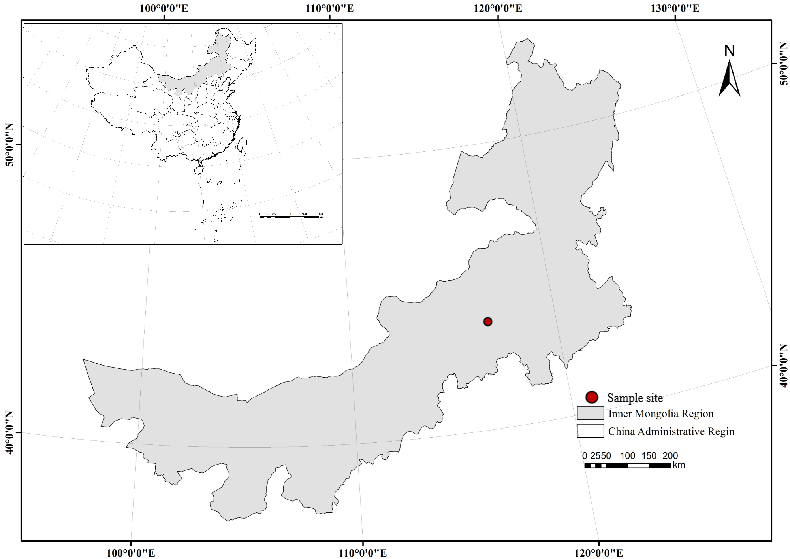


**Figure A2.** (a) Mean annual temperature (MAT) and annual precipitation (AP) and (b) mean monthly temperature and precipitation for the study site in the period 1981-2011.


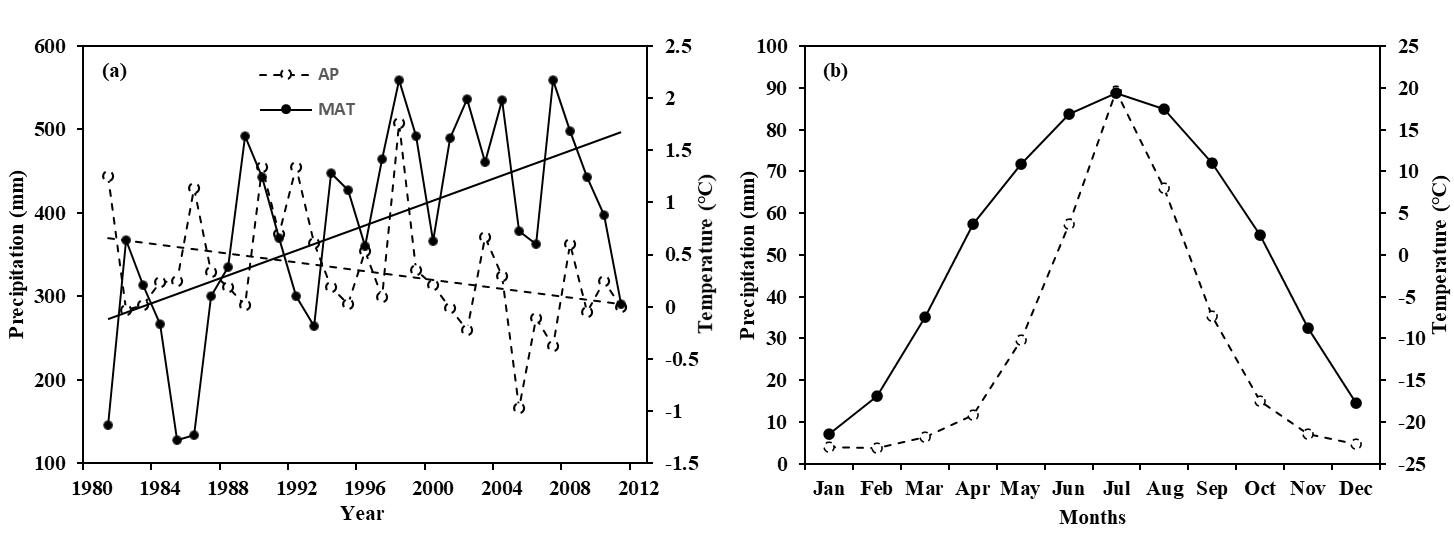


**Table A1.** Results of structural equation modeling of climate variability effects on the community intra-annual stability through all plausible interaction pathways. Given are the standardized path coefficients (estimates), standard error of regression weight (S.E.), the critical value for the regression weight (C.R.), and the level of significance for the regression weight (P). *** indicates P < 0.001.

| Paths | | | Estimate | S.E. | C.R. | P |
| --- | --- | --- | --- | --- | --- | --- |
| Growing season temperature variability | <--- | Growing season precipitation variability | .012 | .367 | .794 | .021 |
| Non-growing season temperature variability | <--- | Non-growing season precipitation variability | .002 | .004 | .233 | .816 |
| Intra-annual species richness | <--- | Growing season precipitation variability | -.038 | .030 | -1.238 | .216 |
| Intra-annual species richness | <--- | Non-growing season precipitation variability | .028 | .020 | 1.411 | .158 |
| Intra-annual species richness | <--- | Growing season temperature variability | -.137 | .055 | -2.489 | .013 |
| Intra-annual species richness | <--- | Non-growing season temperature variability | .012 | .032 | .378 | .706 |
| Community intra-annual biomass | <--- | Growing season precipitation variability | .549 | .356 | 1.542 | .123 |
| Community intra-annual biomass | <--- | Non-growing season precipitation variability | -.375 | .232 | -1.622 | .105 |
| Community intra-annual biomass | <--- | Growing season temperature variability | -1.341 | .698 | -1.922 | .055 |
| Community intra-annual biomass | <--- | Non-growing season temperature variability | -.203 | .367 | -.552 | .581 |
| Community intra-annual biomass | <--- | Intra-annual species richness | 3.016 | 2.238 | 1.347 | .178 |
| Intra-annual species asynchrony | <--- | Growing season precipitation variability | -.006 | .002 | -3.081 | .002 |
| Intra-annual species asynchrony | <--- | Non-growing season precipitation variability | -.001 | .001 | -.499 | .618 |
| Intra-annual species asynchrony | <--- | Growing season temperature variability | .001 | .004 | .236 | .813 |
| Intra-annual species asynchrony | <--- | Non-growing season temperature variability | .003 | .002 | 1.812 | .070 |
| Intra-annual species asynchrony | <--- | Intra-annual species richness | .001 | .012 | .070 | .034 |
| Intra-annual species asynchrony | <--- | Community intra-annual biomass | .002 | .001 | 1.673 | .094 |
| Intra-annual species asynchrony | <--- | Intra-annual biomass stability of PR | .240 | .580 | .413 | .679 |
| Intra-annual species asynchrony | <--- | Intra-annual biomass stability of PB | .320 | .620 | .434 | .532 |
| Intra-annual species asynchrony | <--- | Intra-annual biomass stability of PF | -.106 | .154 | -.687 | .492 |
| Intra-annual species asynchrony | <--- | Intra-annual biomass stability of SS | -.185 | .297 | -.624 | .533 |
| Intra-annual species asynchrony | <--- | Intra-annual biomass stability of AB | -.473 | .346 | -1.368 | .171 |
| Intra-annual species asynchrony | <--- | Dominant species intra-annual biomass stability | .008 | .019 | 0.988 | .035 |
| Intra-annual biomass stability of PB | <--- | Growing season precipitation variability | .004 | .008 | .511 | .609 |
| Intra-annual biomass stability of PB | <--- | Non-growing season precipitation variability | -.008 | .004 | -1.786 | .074 |
| Intra-annual biomass stability of PB | <--- | Growing season temperature variability | .011 | .013 | .794 | .427 |
| Intra-annual biomass stability of PB | <--- | Non-growing season temperature variability | -.009 | .007 | -1.276 | .202 |
| Intra-annual biomass stability of PB | <--- | Intra-annual species richness | .058 | .041 | 1.394 | .163 |
| Intra-annual biomass stability of PB | <--- | Community intra-annual biomass | .002 | .004 | .616 | .538 |
| Intra-annual biomass stability of AB | <--- | Growing season precipitation variability | -.001 | .004 | -.206 | .837 |
| Intra-annual biomass stability of PR | <--- | Growing season precipitation variability | -.002 | .006 | -.384 | .701 |
| Intra-annual biomass stability of PF | <--- | Growing season precipitation variability | .000 | .002 | -.156 | .876 |
| Intra-annual biomass stability of SS | <--- | Growing season precipitation variability | .003 | .003 | .964 | .335 |
| Intra-annual biomass stability of AB | <--- | Non-growing season precipitation variability | .001 | .002 | .311 | .756 |
| Intra-annual biomass stability of PR | <--- | Non-growing season precipitation variability | .003 | .003 | .891 | .373 |
| Intra-annual biomass stability of PF | <--- | Non-growing season precipitation variability | .000 | .001 | -.459 | .646 |
| Intra-annual biomass stability of SS | <--- | Non-growing season precipitation variability | -.004 | .002 | -2.236 | .025 |
| Intra-annual biomass stability of AB | <--- | Growing season temperature variability | -.001 | .007 | -.200 | .842 |
| Intra-annual biomass stability of PR | <--- | Growing season temperature variability | -.005 | .010 | -.474 | .636 |
| Intra-annual biomass stability of SS | <--- | Growing season temperature variability | -.007 | .006 | -1.195 | .232 |
| Intra-annual biomass stability of PF | <--- | Growing season temperature variability | -.003 | .003 | -.912 | .362 |
| Intra-annual biomass stability of AB | <--- | Non-growing season temperature variability | .000 | .004 | -.035 | .972 |
| Intra-annual biomass stability of PR | <--- | Non-growing season temperature variability | -.002 | .005 | -.362 | .717 |
| Intra-annual biomass stability of PF | <--- | Non-growing season temperature variability | -.003 | .002 | -1.795 | .073 |
| Intra-annual biomass stability of SS | <--- | Non-growing season temperature variability | -.003 | .003 | -.941 | .347 |
| Intra-annual biomass stability of PR | <--- | Intra-annual species richness | .002 | .032 | .060 | .952 |
| Intra-annual biomass stability of AB | <--- | Intra-annual species richness | -.008 | .021 | -.404 | .687 |
| Intra-annual biomass stability of PF | <--- | Intra-annual species richness | .018 | .009 | 1.988 | .047 |
| Intra-annual biomass stability of SS | <--- | Intra-annual species richness | .026 | .018 | 1.452 | .147 |
| Intra-annual biomass stability of PR | <--- | Community intra-annual biomass | .001 | .003 | .233 | .816 |
| Intra-annual biomass stability of PF | <--- | Community intra-annual biomass | .002 | .001 | 2.098 | .136 |
| Intra-annual biomass stability of SS | <--- | Community intra-annual biomass | -.001 | .002 | -.913 | .361 |
| Intra-annual biomass stability of AB | <--- | Community intra-annual biomass | .001 | .002 | .320 | .749 |
| Dominant species intra-annual biomass stability | <--- | Growing season precipitation variability | .005 | .005 | 1.173 | .241 |
| Dominant species intra-annual biomass stability | <--- | Non-growing season precipitation variability | .000 | .003 | .022 | .983 |
| Dominant species intra-annual biomass stability | <--- | Growing season temperature variability | -.013 | .008 | -1.553 | .120 |
| Dominant species intra-annual biomass stability | <--- | Non-growing season temperature variability | -.005 | .004 | -1.124 | .261 |
| Dominant species intra-annual biomass stability | <--- | Intra-annual species richness | .056 | .026 | 2.158 | .061 |
| Intra-annual biomass stability of PR | <--- | Dominant species intra-annual biomass stability | .431 | .154 | 2.802 | .015 |
| Intra-annual biomass stability of PF | <--- | Dominant species intra-annual biomass stability | -.256 | .763 | -.336 | .737 |
| Intra-annual biomass stability of PB | <--- | Dominant species intra-annual biomass stability | .052 | .166 | .314 | .753 |
| Dominant species intra-annual biomass stability | <--- | Community intra-annual biomass | -.003 | .002 | -1.378 | .168 |
| Community intra-annual biomass stability | <--- | Intra-annual biomass stability of SS | .624 | .497 | 1.255 | .209 |
| Community intra-annual biomass stability | <--- | Intra-annual biomass stability of PB | 1.191 | .290 | 4.108 | .058 |
| Community intra-annual biomass stability | <--- | Intra-annual biomass stability of PR | .009 | .306 | .028 | .007 |
| Community intra-annual biomass stability | <--- | Intra-annual biomass stability of PF | 2.816 | 1.332 | 2.115 | .074 |
| Community intra-annual biomass stability | <--- | Dominant species intra-annual biomass stability | .523 | .341 | 1.532 | .005 |
| Community intra-annual biomass stability | <--- | Intra-annual species asynchrony | 5.555 | .959 | 5.792 | *** |
| Community intra-annual biomass stability | <--- | Intra-annual biomass stability of AB | -.298 | .423 | -.703 | .482 |
| Community intra-annual biomass stability | <--- | Intra-annual species richness | .088 | .049 | 1.781 | .045 |
| Community intra-annual biomass stability | <--- | Community intra-annual biomass | -.007 | .004 | -1.571 | .116 |
| Community intra-annual biomass stability | <--- | Growing season precipitation variability | -.014 | .008 | -1.713 | .087 |
| Community intra-annual biomass stability | <--- | Non-growing season precipitation variability | .009 | .005 | 1.839 | .066 |
| Community intra-annual biomass stability | <--- | Growing season temperature variability | .018 | .015 | 1.185 | .236 |
| Community intra-annual biomass stability | <--- | Non-growing season temperature variability | .015 | .008 | 2.022 | .153 |

**Table A2.** Common species and their relative abundance during the long-term observation period from 1981 to 2011.

| Species |  | Functional group |  | Relative abundance |  | Species |  | Functional group |  | Relative abundance |
| --- | --- | --- | --- | --- | --- | --- | --- | --- | --- | --- |
| *Leymus chinensis* |  | PR |  | 25.5 ± 4.2 % |  | *Stipa*  *grandis* |  | PB |  | 19.1 ± 3.6 % |
| *Agropyron cristatum* |  | PR |  | 7.1 ± 1.6 % |  | *Achnatherum sibiricum* |  | PB |  | 11.2 ± 2.7 % |
| *Carex korshinskii* |  | PR |  | 2.9 ± 1.2 % |  | *Cleistogenes squarrosa* |  | PB |  | 1.92 ± 1.1 % |
| *Heteropappus altaicus* |  | PF |  | 0.73 ± 0.7 % |  | *Poa*  *attenuata* |  | PB |  | 0.74 ± 0.9 % |
| *Allium anisopodium* |  | PF |  | 0.65 ± 0.6 % |  | *Koeleria*  *macrantha* |  | PB |  | 0.34 ± 0.4 % |
| *Pulsatilla*  *turczaninovi* |  | PF |  | 0.47 ± 0.3 % |  | *Thalictrum petaloideum* |  | PF |  | 0.99 ± 0.7 % |
| *Medicago ruthenica* |  | PF |  | 0.21 ± 0.3 % |  | *Artemisia pubescens* |  | PF |  | 0.28 ± 0.2 % |
| *Phlomis mongolica* |  | PF |  | 0.22 ± 0.4 % |  | *Haplophyllum dauricum* |  | PF |  | 0.31 ± 0.3 % |
| *Oxytropis myriophylla* |  | PF |  | 0.82 ± 1.0 % |  | *Potentilla bifurca* |  | PF |  | 0.94 ± 0.9 % |
| *Limonium bicolor* |  | PF |  | 0.24 ± 0.08 % |  | *Allium condensatum* |  | PF |  | 0.48 ± 0.1 % |
| *Saposhnikovia divaricata* |  | PF |  | 0.28 ± 0.09 % |  | *Potentilla tanacetifolia* |  | PF |  | 0.84 ± 0.5 % |
| *Glycyrrhiza uralensis* |  | PF |  | 0.27 ± 0.04 % |  | *Potentilla verticillaris* |  | PF |  | 0.81 ± 0.4 % |
| *Tephroseris kirilowii* |  | PF |  | 0.30 ± 0.07 % |  | *Klasea centauroides* |  | PF |  | 0.78 ± 0.6 % |
| *Leontopodiu leontopodioides* |  | PF |  | 0.31 ± 0.07 % |  | *Pedicularis striata* |  | PF |  | 0.58 ± 0.2 % |
| *Linariavulgaris subsp. sinensis* |  | PF |  | 0.38 ± 0.04 % |  | *Astragalus galactites* |  | PF |  | 0.62 ± 0.3 % |
| *Galium verum* |  | PF |  | 0.31 ± 0.07 % |  | *Adenophora stenanthina* |  | PF |  | 0.57 ± 0.3 % |
| *Thermopsis lanceolata* |  | PF |  | 0.32 ± 0.04 % |  | *Allium senescens* |  | PF |  | 0.52 ± 0.2 % |
| *Euphorbia esula* |  | PF |  | 0.26 ± 0.05 % |  | *Allium tenuissimum* |  | PF |  | 0.91 ± 0.6 % |
| *Sibbaldia adpressa* |  | PF |  | 0.31 ± 0.06 % |  | *Iris*  *tenuifolia* |  | PF |  | 0.72 ± 0.4 % |
| *Allium*  *bidentatum* |  | PF |  | 0.31 ± 0.02 % |  | *Potentilla acaulis* |  | PF |  | 0.91 ± 0.3 % |
| *Phedimus aizoon* |  | PF |  | 0.29 ± 0.01 % |  | *Linum*  *perenne* |  | PF |  | 0.41 ± 0.1 % |
| *Bupleurum scorzonerifolium* |  | PF |  | 0.20 ± 0.03 % |  | *Allium ramosum* |  | PF |  | 0.52 ± 0.2 % |
| *Gueldenstaedtia verna* |  | PF |  | 0.18 ± 0.02 % |  | *Astragalus adsurgens* |  | PF |  | 0.46 ± 0.2 % |
| *Cymbaria daurica* |  | PF |  | 0.19 ± 0.03 % |  | *Nepeta multifida* |  | PF |  | 0.57 ± 0.2 % |
| *Silene jenisseensis* |  | PF |  | 0.34 ± 0.06 % |  | *Silene*  *aprica* |  | PF |  | 0.38 ± 0.1 % |
| *Saussurea japonica var pteroclada* |  | PF |  | 0.21 ± 0.04 % |  | *Caragana microphylla* |  | SS |  | 3.39 ± 0.9 % |
| *Artemisia frigida* |  | SS |  | 2.41 ± 0.6 % |  | *Kochia prostrata* |  | SS |  | 0.7 ± 0.3 % |
| *Dysphania aristata* |  | AB |  | 0.23 ± 0.2 % |  | *Orostachys fimbriatus* |  | AB |  | 0.3 ± 0.1 % |
| *Chenopodium album* |  | AB |  | 0.67 ± 0.7 % |  | *Dontostemon micranthus* |  | AB |  | 0.68 ± 0.5 % |
| *Gentiana squarrosa* |  | AB |  | 0.42 ± 0.2 % |  | *Axyris amaranthoides* |  | AB |  | 0.61 ± 0.4 % |
| *Salsola collina* |  | AB |  | 1.39 ± 0.4 % |  |  |  |  |  |  |

Abbreviations: perennial rhizome grass (PR), perennial bunchgrasses (PB), perennial forbs (PF), shrubs and semi-shrubs (SS), and annuals and biennials (AB).
